# Collaborative Problem Solving: Processing Actions, Time, and Performance

**DOI:** 10.3389/fpsyg.2019.01280

**Published:** 2019-06-07

**Authors:** Paul De Boeck, Kathleen Scalise

**Affiliations:** ^1^Department of Psychology, The Ohio State University, Columbus, OH, United States; ^2^Department of Psychology, KU Leuven, Leuven, Belgium; ^3^Department of Educational Methodology, Policy, and Leadership, University of Oregon, Eugene, OR, United States

**Keywords:** problem solving, strategy, factor model, measurement, collaboration

## Abstract

This study is based on one collaborative problem solving task from an international assessment: the Xandar task. It was developed and delivered by the Organization for Economic Co-operation and Development Program for International Student Assessment (OECD PISA) 2015. We have investigated the relationship of problem solving performance with invested time and number of actions in collaborative episodes for the four parts of the Xandar task. The parts require the respondent to collaboratively plan a process for problem solving, implement the process, reach a solution, and evaluate the solution (For a full description, see the Materials and Methods section, “Parts of the Xandar Task.”) Examples of an action include posting to a chat log, accessing a shared resource, or conducting a search on a map tool. Actions taken in each part of the task were identified by PISA and recorded in the data set numerically. A confirmatory factor analysis (CFA) model looks at two types of relationship: at the level of latent variables (the factors) and at extra dependencies, which here are direct effects and correlated residuals (independent of the factors). The model, which is well-fitting, has three latent variables: actions (A), times (T), and level of performance (P). Evidence for the uni-dimensionality of performance level is also found in a separate analysis of the binary items. On the whole for the entire task, participants with more activities are less successful and faster, based on the United States data set employed in the analysis. By contrast, successful participants take more time. By task part, the model also investigates relationships between activities, time, and performance level within the parts. This was done because one can expect dependencies within parts of such a complex task. Results indicate some general and some specific relationships within the parts, see the full manuscript for more detail. We conclude with a discussion of what the investigated relationships may reveal. We also describe why such investigations may be important to consider when preparing students for improved skills in collaborative problem solving, considered a key aspect of successful 21st century skills in the workplace and in everyday life in many countries.

## Introduction

The construct explored here, collaborative problem solving (CPS), was first introduced to the Program for International Student Assessment (PISA) in 2015. Attempts to explore process data collected in complex activities such as CPS are emerging rapidly in education. Yet which models might best fit process data and the analytic techniques to employ to investigate patterns in the data are not well understood at this time. So here we investigate whether relationships seen in the actions taken by PISA respondents, as coded by PISA, might shed light on approaches for modeling complex CPS tasks.

In the CPS task released by PISA, the Xandar task, there are four parts. The parts of the task require the respondent to collaborate to plan a process for problem solving, implement the process, reach a solution, and evaluate the solution. (For a full description of these parts, see the Materials and Methods section, “Parts of the Xandar Task.”) Examples of actions in Part 1, for instance, include posting to a chat log, accessing a shared resource, or conducting a search on a shared map tool.

In each of the parts, process data are available on time spent and number of actions, as well as on the performance on specific items within the four parts. We explore modeling these Xandar data to address three research questions:

RQ1. Does a factor model employing process data (actions and time) support evidence for a latent variable differentiation between the types of process data (actions, time) and between the latter two and quality of performance? The expected latent variables are Actions, Time, and Performance.
RQ2. Do extra dependencies at the level of the observed variables improve model fit, including direct effects and correlated residuals (independent of the factors)? If they do, they reveal direct relationships between process aspects and performance, independent of the latent variables. These direct relationships are indications of the dynamics underlying collaborative problem solving, whereas the latent variables and their correlations inform us about global individual differences in process approaches and performance.
RQ3. Can the performance also be considered as uni-dimensional at the specific level of the individual items (from all four Xandar parts)?

In this Xandar investigation, each factor (latent variable) is composed of four corresponding measures from the four Xandar parts. Data are fit with a latent variable model to answer RQ1. Dependencies within parts can be expected between the three measures. So we address the extra dependencies in RQ2. The dependencies are not only considered for methodological reasons when variables stem from the same part, but they may also reveal how subjects work on the tasks. Finally, because a good-fitting factor model would imply uni-dimensionality of the performance sum scores from the four parts, we also explore uni-dimensionality at the level of the individual items in RQ3.

Sections in this paper first discuss the PISA efforts to explore problem solving in 2012 and 2015 assessments, then offer a brief summary of the literature on CPS. Next in the Materials and Methods section, we discuss the PISA 2015 collaborative complex problem solving released task, “Xandar,” including the availability of the released code dictionary and data set. In the Results and Discussion, we model United States data from the Xandar task and report results to address the three research questions.

## PISA and a Brief Summary of Literature on CPS

The PISA 2015 CPS construct, which included measuring groups in collaboration, was built on PISA’s 2012 conception of individual problem solving (OECD, 2014). In PISA 2012, some student individual characteristics related to individual problem solving were measured. These measures were openness to learning, perseverance, and problem solving strategies.

For the 2015 PISA collaborative framework (OECD, 2013), the construct of problem solving was extended from 2012 in order to include measures of group collaboration. For this new assessment in 2015, it was recognized that the ability of an individual to be successful in many modern situations involves participating in a group. Collaboration was intended to include such challenges as communicating within the group, managing conflict, organizing a group, and building consensus, as well as managing progress on a successful solution.

The PISA framework described the importance of improving collaboration skills for students (Rummel and Spada, 2005; Vogel et al., 2016) The measurement of collaboration skills was at the heart of problem solving competencies in the PISA CPS 2015 framework. The framework specified first that the competency being described remained the capacity of an individual, not the group. Secondly, the respondent must effectively engage in a process whereby two or more agents attempt to solve a problem, where the agents can be people or simulations. Finally, the collaborators had to show efficacy by sharing the understanding and effort required to come to a solution, such as pooling knowledge to reach solutions.

Approaches to gathering assessment evidence cited by the PISACPS framework (OECD, 2013) ranged from allowing *actions* during collaboration to evaluating the *results* from collaboration. Measures of collaboration in the research literature include solution success, as well as processes during the collaboration (Avouris et al., 2003). *In situ* observables for such assessments could include analyses of log files in which the computer keeps a record of student activities, sets of intermediate results, and paths taken along the way (Adejumo et al., 2008). Group interactions also offer relevant information (O’Neil et al., 1997), including quality and type of communication (Cooke et al., 2003; Foltz and Martin, 2008; Graesser et al., 2008) and judgments (McDaniel et al., 2001).

The international Assessment and Teaching for twenty-first century Skills (ATC21S) project also examined the literature on disposition to collaboration and to problem solving in online environments. ATC21S described how interface design feature issues and the evaluation of CPS processes interact in the online collaboration setting (Scalise and Binkley, 2009; Binkley et al., 2010, 2012).

In the PISA 2015 CPS assessment, a student’s collaborative problem-solving ability is assessed in scenarios where the student must solve a problem. For collaboration, the problem is solving working with “agents,” or computer avatars that simulate collaboration. The CPS framework describes that a problem need not be subject-matter specific task,. Rather it could also be as a partial task in an everyday problem. Examples of subject-matter specific problem solving include setting up a sustainable fish farm in science, planning the construction of a bridge using engineering and mathematics, or writing a persuasive letter using language arts and literacy Examples of an “everyday” problem include communicating with others to delegate roles during collaboration for event planning, monitoring to ensure a group remains on task, and evaluating whether collaboration is complete. All these actions can be directed toward the ultimate goal.

In the PISA 2015 perspective, assessment is continuous throughout the unit and can incorporate student’s interactions with the digital agents. Each student response on a traditional question follows a stream of actions during which the student has chosen how to interact and collaborate with standardized agents in each particular task situation. Very few of the collaborative actions and tasks are released by PISA, but the *number* of collaborative actions in each part of the task are released and made available in the PISA data sets. So here we accept that PISA has coded the action as taking place, and analyze the numeric results provided.

## Materials and Methods

### Parts of the Xandar Task

Here we analyze numeric data provided for the PISA 2015 Xandar unit (OECD, 2017a, 2017b). In the unit Xandar:

“A three-person team consisting of the student test-taker and two computer agents takes part in a contest where [the team] must answer questions about the fictional country of Xandar. The questions [involve] Xandar’s geography, people and economy. This unit involves decision-making and coordination tasks, requires consensus-building collaboration, and has an in-school, private, and non-technology-based context.”

Xandar is a fictional planet appearing in comic books published by Marvel Comics. In the PISA Xandar task, it is treated as a mythical location to be investigated collaboratively. The Xandar task has four parts:

• Part 1 – Agreeing on a Strategy. This part of the Xandar activity familiarizes the student with how the contest will proceed, the chat interface and the task space including buttons that students can click to take actions in particular situations and a scorecard that monitors team progress. In Part 1, the student is assigned to work in a team with digital agents named Alice and Zach. A variety of actions are available. The respondent and the agents interact to generate a stream of actions. The respondent is expected to follow the rules of engagement provided for the contest and to effectively establish collaborative and problem-solving strategies that were the goal of Part 1.
• Part 2 – Reaching a Consensus Regarding Preferences. In this part of the Xandar activity, group members should take responsibility for the contest questions in one subject area (Xandar’s geography, people, or economy). The team members must apportion the subject areas among themselves. The agents begin by disagreeing. The student has opportunities to help resolve the disagreement, can take a variety of actions, and the goal is to establish common understanding.
• Part 3 – Playing the Game Effectively. In this part of the Xandar activity, group members begin playing the game by answering geography contest questions together. The group has the opportunity to choose among answers, during which the agents interject questions, pose concerns and even violate game rules. The student exhibits collaborative problem solving strategies through actions and responses.
• Part 4 – Assessing Progress. In this part of the Xandar activity, agent Alice has posed a question about its progress. The student responds with an evaluation. Regardless of the student’s answer, agent Zach indicates he is experiencing trouble information foraging for his assigned subject area, economy. Responses and actions take place regarding both evaluating and supporting group members.

Each of the four parts comes with a number of items to score the performance. The complete Xandar released task is presented in an OECD PISA report that illustrates the items that students faced in the 2015 PISA collaborative problem-solving assessment (OECD, 2016). The released code dictionary and data are also available on the 2015 PISA website. We do not repeat the Xandar information here (due in part to copyright), but summarize only. The Xandar released unit presents:

• a screenshot of each item
• the correct action(s) or response to the item
• an explanation as to why the action or response is correct
• the skills that are examined by the item
• alignments describing the difficulty of the item.

### Sample

As described earlier, this study employed data publicly released from the Organization for Economic Co-operation and Development Program for International Student Assessment (OECD PISA) for the optional collaborative problem solving (CPS) assessment. It was administered in 2015 to nationally representative samples of approximately age 15 students. Since PISA is designed to have systematically missing data in a matrix sample, only students who took the Xandar task were included. Students were sampled according to the PISA sample frame. Data analyzed here are representatively sampled United States participants from the Xandar released task. See Table 1 for descriptives by age, gender and race/ethnicity of the United States Xandar task sample used.

**TABLE 1:** Descriptives for collaborative problem solving Xandar assessment for the United States sample.

**Descriptive**	**N**	**Percentage**
Total sample	986	100%
**Birth year**		
1999	479	48.58%
2000	498	50.51%
Missing	9	<1%
**Gender (binary only in PISAB)**		
Male	503	51.01%
Female	474	48.07%
Missing	9	<1%
**Race/Ethnicity**		
White, not Hispanic	409	41.48%
Black or African American	138	14.00%
Hispanic or Latino	314	31.85%
Asian	36	3.65%
Multi-racial	67	6.80%
Other	7	<1%
Missing	15	1.52%

From the 994 students who took the Xandar task, 986 have complete Xandar data. The descriptive statistics and all analyses are based on *N* = 986. (Note that limitations to be discussed later in this manuscript include only United States data examined to date in this exploration. Extensions to more countries and comparisons across countries are an exciting and interesting potential to the work. However, the international extensions are out of scope for this article.) For the purposes of the current study, the school variable was not employed. All students were treated as one group.

Regarding ethical approval and consent for human subjects data collection in PISA, OECD gains ethical approval and consent through PISA processes. Processes are established in coordination with each country for public release of some de-identified data collected in PISA main study assessments. Data sets made available for release are intended for purposes of secondary research. The CPS data set used here is available through the OECD data repository website^1^.

As discussed earlier, for the Xandar task, released data are available for actions, time and level of performance. The data for the current study included four indicators each of CPS actions taken (parts 1–4), time taken (parts 1–4), and success scores (parts 1–4). These become the three latent traits, or factors, in this study. To measure CPS actions, we used number of collaboration actions as measured by the data provided in the log transformation of C1A, C2A, C3A, and C4A. “C” indicates this was a collaborative assessment, the numeral indicates the Xandar part, and “A” indicates number of actions taken. To measure timing, we used timing as measured by data provided in the log transformation of C1T, C2T, C3T, and C4T. “C” indicates this was a collaborative assessment, the numeral indicates the Xandar part, and T indicates time taken. To measure student success, we used the sum of the binary item response success scores for each of the four parts, C1P, C2P, C3P, and CP4 (based on 5, 3, 2, and 2 items within the Xandar parts).

Exploratory data analysis following log transformation as described above for some variables revealed only minor deviations from normality. Skewness between −2 to 2 was used for all observed variables (Cohen et al., 2002). Note, however, that this is not a strongly conservative range, as discussed in the limitations. So we also report for this study skewness with all observed variables approximately in the range −1 to 1 except for C1A (1.52) and C2A (1.48). Due to no major levels of deviation, the analysis proceeded without further transformation to the observed variables. Other descriptives for all observed variables are provided in Table 2.

**TABLE 2:** Descriptives for observed variables.

**Variable**	**Mean**	**SD**	**Min**	**Max**	**% Missing**
C1T	11.70	0.30	10.85	12.87	0.00
CIA	2.58	0.33	0.00	5.12	0.00
C2T	11.20	0.29	9.52	13.17	0.00
C2A	2.14	0.28	0.00	3.74	0.00
C3T	11.19	0.31	9.71	12.17	0.00
C3A	2.76	0.39	0.00	4.03	0.00
C4T	10.19	0.45	8.78	11.51	0.00
C4A	1.61	0.27	0.69	3.58	0.00
C1P	3.49	1.35	0	5	0.00
C2P	1.95	0.86	0	3	0.00
C3P	1.03	0.56	0	2	0.00
C4P	0.99	0.74	0	2	0.00

We fit the model using lavaan (Rosseel, 2012) in R version 3.5.1 (R Core Team, 2018). We used the weighted least squares “WLSMV” option which employs the diagonally weighted least squares (DWLS) estimator with robust standard errors and a mean and variance adjusted test statistic. We have estimated a confirmatory factor analysis (CFA) model with three factors (each with standardized latent variables). The factors are Actions, Time, and Performance. Each one has the four corresponding measures from the four Xandar parts.

Because dependencies within parts can be expected between the three measures, some parameters were added to the model. They are direct within-part effects of actions on time (more actions implies more time), direct within-part effects of performance on time (better performance may take more time), and correlated residuals for actions and performance within each part (exploring the relationship between actions and performance level).

Direct effects and residual correlations are two different types of dependencies. Direct effects are effects of one variable on another (e.g., of $Y_{1}$ on $Y_{2}$). The two directions, $Y_{1}\to Y_{2}$ and $Y_{2}\to Y_{1}$, are not mathematically equivalent. Correlated residuals are equivalent with the effect of a residual of one variable on the other variable (e.g., of $\varepsilon_{Y\,1}$ on $Y_{2}$). the two directions are mathematically equivalent and equivalent with the covariance of the residuals. To be clear, neither of the dependencies prove a causality relation. A causal hypothesis can be at the basis of hypothesizing a direct effect, whereas correlated residuals can be used for explorative purposes, without specifying a direction. For the present study, we hypothesized that more actions take more time and that a higher level of performance requires more time. For number of actions and level of performance we explore the dependency with correlated residuals.

**FIGURE 1:**
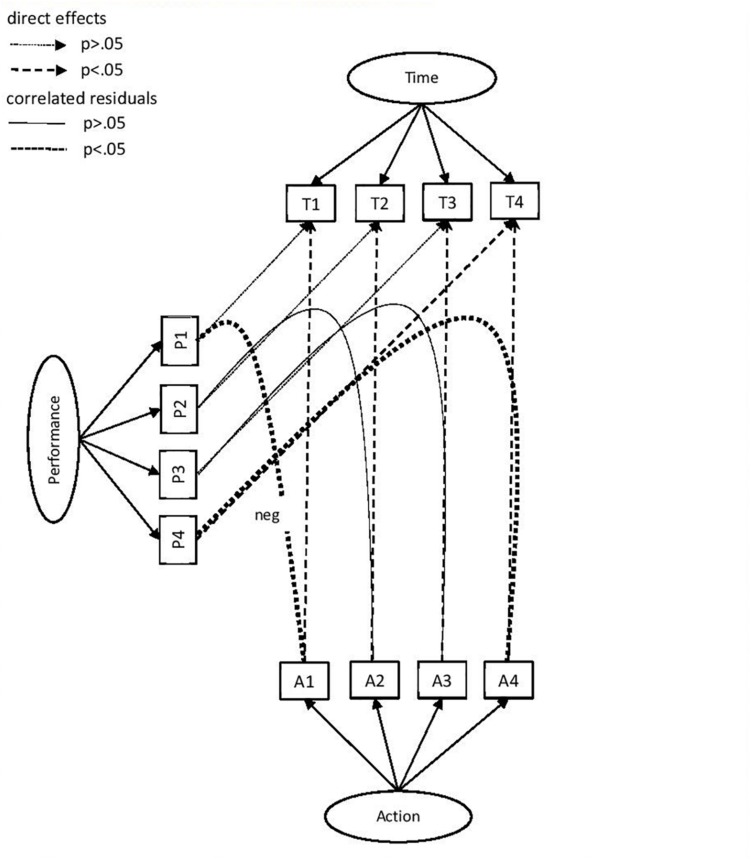
Latent variable and dependency model for Xandar data. The latent variables are Time, Actions, and Performance. The observed variables per factor are indicated with capital letters referring to the latent variable (T, A, P) and with a number referring to the Xandar part (1, 2, 3, and 4). The direct effects between observed variables from the same Xandar part are indicated with single headed dashed arrows (between the A and T and between the P and T). The correlated residuals are indicated with dotted lines without arrow. Significance (*p*<–01) is denoted with a thicker dashed arrow (direct effects) or line (correlated residuals). All dependencies are positive except when indicated with “neg” (between Al and PI). Correlations between latent variables, factor loadings, residual variances, and dependency values are omitted to avoid clutter in the figure. The correlations between the latent variables can be found in the text, the factor loadings are presented in Table 3, and the dependency values in Table 4.

See the row heads of Tables 3, 4 and Figure 1 for a definition of the model estimated. It includes the latent variable structure as well as the dependencies. The model can also be derived from the R code for the analysis, which is available in the Supplementary Material.

**TABLE 3:** CFA factor loadings Xandar measures.

**Variable**	**Estimate**	**SE**	***z***	***p***	**Standardized**
**Action factor**					
CIA	0.19	0.02	7.84	<0.001	0.57
C2A	0.17	0.02	7.61	<0.001	0.63
C3A	0.22	0.03	7.91	<0.001	0.58
C4A	0.05	0.01	4.09	<0.001	0.19
**Time factor**					
C1T	0.26	0.01	20.48	<0.001	0.85
C2T	0.24	0.01	17.71	<0.001	0.84
C3T	0.17	0.01	13.20	<0.001	0.54
C4T	0.19	0.02	11.87	<0.01	0.43
**Performance factor**					
C1P	0.87	0.05	16.81	<0.01	0.64
C2P	0.47	0.03	14.09	<0.01	0.55
C3P	0.25	0.02	10.58	<0.01	0.44
C4P	0.28	0.03	10.39	<0.01	0.38

**TABLE 4:** Extra dependencies in CFA model for Xandar measures.

**Variables**	**Estimate**	**SE**	***z***	***p***	**Standardized**
CIA→CIT	0.41	0.047	8.57	<0.001	0.45
C2A→C2T	0.52	0.084	6.11	<0.001	0.49
C3A→C3T	0.30	0.046	6.56	<0.001	0.37
C4A→C4T	0.49	0.061	7.94	<0.001	0.29
CIP→CIT	0.01	0.01	1.10	>0.05	0.04
C2P→C2T	0.00	0.03	–0.21	>0.05	–0.01
C3P→C3T	0.00	0.02	–0.43	>0.05	–0.01
C4P→C4T	0.26	0.02	15.28	<0.001	0.42
CIP→CIA	–0.04	0.01	–3.14	<0.01	–0.16
C2P↔C2A	0.01	0.81	0.42	>0.05	0.04
C3P↔C3A	0.01	0.88	0.38	>0.05	0.04
C4P↔C4A	0.06	0.01	9.67	<0.001	0.32

*→indicates direct effects and ↔ indicates correlated residuals.*

## Results

In this section we describe the results of the modeling. With the dependencies as described in the Methods section added to the model, the model fit was good (close), with a TLI of 0.95 and RMSEA of 0.038 (90% CI 0.029 to 0.048). Without the dependencies (without the eight direct effects and four residual correlations), the model fit is clearly worse, with a TLI of 0.574 and RMSEA of 0.112 (90% CI 0.104 to 0.119). These results address RQ1 and RQ2.

The correlations between the latent variables are −0.473, *p*<0.001 (Actions and Time), −0.732, *p* < 0.001 (Actions and Performance), and 0.190, *p* < 0.01 (Time and Performance). The loadings and dependencies are shown in Tables 3, 4, respectively. As expected, the indicators of actions, time, and performance all showed significant positive factor loadings on the corresponding factors (see Table 3). The standardized coefficients in the last column indicate that the loadings of the Part 4 indicators are lower than those of the other three parts: 0.19 (Actions), 0.43 (Time), and 0.38 (Performance).

Table 4 shows the estimates of the dependencies:

• Number of activities makes time longer: a significant positive effect was found for all four parts.
• A significant positive effect of performance on time was found only for Part 4. For the other parts the effect was almost zero.
• Number of activities and performance levels have significant correlated residuals for two parts. For explorative reasons the dependencies were not tested with a direction but with correlated residuals instead. The results were found to be different depending on the part. Results showed negative dependency for Part 1, a positive dependency for Part 4, and an almost zero dependency for the Parts 2 and 3.

Although the factor model with these dependencies fits well, we wanted to check whether the performance is also uni-dimensional at the level of the individual items (RQ3). Uni-dimensionality of the four sum scores as implied by the factor model, does not imply uni-dimensionality at the level of the 12 individual binary items. This is especially because the items represent four processes (exploring and understanding, representing and formulating, planning and executing, and monitoring and reflecting) and three competencies (establishing and maintaining shared understanding, taking appropriate action to solve the problem, and establishing and maintaining team organization), but not with a perfectly crossed design.

The answer to the dimensionality question based on the analysis with this data set is that the 12 items can be considered as uni-dimensional based on the empirical data, although they are designed to tap on a diversity of processes and competencies. The uni-dimensional model fit was good (close), with a TLI of 0.94 and RMSEA of 0.037 (90% CI 0.029, 0.046). The uni-dimensional model is the result of an ordinal confirmatory factor model for the binary items using WLSMV and the same lavaan version as for the earlier analysis. For the delta parameterization the loadings vary between 0.272 and 0.776 and they are all significant (*p* < 0.001).

## Discussion

For the model with loadings and dependencies showing in Tables 3, 4, the latent variable correlations of Actions with Time and with Performance are negative. Hence, participants showing more activities are faster and perform less well in their collaborative problem solving. This is based on the United States dataset with the Xandar task. Successful participants take more time, perhaps a consequence of the previous two relationships. Multiplying the two negative correlations yields −0.473 × −0.723 = 0.346, which is higher than the 0.190 estimate of the correlation between Time and Performance. This explains that in an alternative but formally equivalent model with an effect of Actions on Time and on Performance, the correlation between the residuals of the latent variables Time and Performance is negative. However, the correlation of −0.260 in question is not significant (*p* > 0.05).

The negative correlation between Actions and Time suggests that highly active students are fast and not so active students are slow. The combination of fast and active on the latent variables seem to reflect an impulsive and fast trial-and-error style. This strategy shows itself in the Xandar task as not very successful versus a slower, more thoughtful and apparently more successful style. It makes sense that respondents who are more deliberative may have more knowledge to bring to considering a successful solution, or be exhibiting more test effort in the Xandar context. We do not have the information to examine what is happening during the deliberation. This is in part because descriptions of the possible actions are not available in the data set. As well there is no interpretive information provided by PISA for the sample. This could include think-alouds where students describe why they are doing what they are doing. It could also have included qualitative response process information in which student explain their processes, in-depth interviews, or other approaches that supply interpretive information.

However, it makes of course sense that more actions take more time, which shows in the analysis of the dependencies between observed actions and time. This illustrates why it is informative to differentiate relationships between latent variables from relationships which show in dependencies.

Other important dependencies concern Part 4, which is a clearly reflective task, a kind of reflective and evaluative pause. The nature of the task may explain why performance is associated with more actions and requires more time, in contrast with Part 1 (agreeing on a strategy) where the association between actions and performance is negative. For instance, too much discussion on a strategy may signal a lack of structure.

For the result that the items examined can be considered as uni-dimensional although they are designed to tap on a diversity of processes and competencies, this suggests that the collaborative ability generalizes across processes. In other words, the collaborative competencies rely on a general underlying ability. The specificities of the processes are reflected in the extra dependencies. Part 4 involves monitoring and reflecting. This may explain why more activities and more time are associated with better performance. Part 1 by contrast involves planning and execution and representing and formulating. This may lead to better results if not based on trial and error (many actions) but on a structured and goal-oriented approach (less actions).

These dependencies suggest that, depending on the task, the collaborative ability may rely on a general underlying ability but be implemented through a different approach in various collaborative actions, as has been discussed in the literature (Fiore et al., 2017; OECD, 2017b; Eichmann et al., 2019). The special and specific status of Part 4 is also reflected in its lower loadings on all three latent variables (see standardized loadings).

Note that the extra dependencies here are not only considered for methodological reasons when variables stem from the same part. They may also reveal how subjects work on the tasks. This is consistent with the findings here. Parts such as 1 and 4 have a distinct theoretical description in the PISA framework. But how they draw on the collaborative ability can be seen in the empirical data to seemingly require different approaches as indicated in the process data.

Taken together, these results for the United States data set are consistent with problem solving performance modeled as invested time and number of actions.

Potential impacts underscore that it seems possible both to collect and to scale information on the collaborative ability. Measures may help provide intervention support, since in today’s world especially, teams with good collaborative skills are necessary in any group. Groups can range from families to corporations, public institutions, organizations, and government agencies (OECD, 2013). Previously, dispositions to collaborate were reported based on the PISA data (Scalise et al., 2016). Indicators of collaborative ability also may be needed to create adequate interventions to train collaboration skills and to change current levels of individual collaboration.

As previously reported, the disposition dimensions of *collaborate*, *negotiate*, and *advocate*/*guide* might be useful starting points for creating such interventions (Scalise et al., 2016; OECD, 2017a). Alternatively, the factor structure here may yield suggestions on additional interesting starting points. This could include structures by which a student may approach collaboration (OECD, 2017b; Wilson et al., 2017) but more interpretive information would be needed. This could be combined with how participatory a student is disposed to be in collaboration, along with his or her team leadership inclinations, and beliefs in the value or efficacy of collaboration (Scalise et al., 2016).

Limitations to the analysis here include that only the United States data set of many countries available in the PISA data was analyzed. So this analysis should be extended to more countries and results compared in future work.

Also, from a statistical standpoint as discussed earlier, missing data were excluded listwise. In addition, minor but not major skewness was seen in two of the observed variables. Finally, multilevel modeling was not employed so the nested nature of students within schools was not taken into account.

TLI and RMSEA were reported here as the two fit indices since they seem most commonly used in the educational assessment field for large scale analyses. But there have been limited considerations for CPS on this topic.

For limitations from a conceptual standpoint, OECD releases a limited range of information, for instance items for only one of the 2015 collaborative problem solving tasks (Xandar) was released and collaborative actions were numbered but not described in the data set and data dictionary.

For implications of future work from this study, there are several. First, the era of analyzing process data and not only item response data in robust assessment tasks is upon us (many researchers including Praveen and Chandra, 2017). Approaches such as used here could be applied for other constructs, not just problem solving. Models can consider how to explore two types of relationship:

• at the level of general individual differences (the factors)
• at extra dependencies, which are direct effects and correlated residuals (independent of the factors)

These extra dependencies may provide a window on the underlying process dynamics, see Figure 1. It should be noted for implications for future work that it would be helpful if a range of simplified visualizations could be developed for such complex analyses. Standard plots after including dependencies seemed too complex to be fully useful.

For extensions to the specific modeling here, it would be important as discussed earlier to explore fitting the same or similar models across data sets from other countries (Thomas and Inkson, 2017). This could be augmented by also modeling potential country-level effects at the item level, by exploring differential item functioning. Furthermore it would be interesting to consider covariates available in the PISA student questionnaire data set (SQ) in relation to the collaborative ability examined here. This could include indicators for dispositions for collaborative problem solving that moved forward to the main PISA study (Scalise et al., 2016). These indicators include student-level indicators available in the CPS SQ data set regarding self-report of dispositions toward cooperation, guiding, and negotiating.

It should also be mentioned that other very interesting student-level indicators regarding additional preferences in collaboration had to be dropped from the PISA main study. This was due to time limitations. Dropped indicators included dispositions toward collaborative *leadership*, as well as student-level indicators of in-school and out-of-school collaborative *opportunities*. While these were not possible to include in the main study due to time limitations for the PISA administration, the indicators were part of the field testing. They could be very interesting to administer at the country-level in other national or international assessments.

Teacher-level indicators are also available in the PISA data set that provide information on opportunity to learn (OtL) for students in the PISA CPS. Data include classroom-level OtL reports of team activities, grouping practices, types of collaborative activities, and types of rewards provided for engaging in successful team work. Exploring relationships here might allow more reflection on connections to potential interventions. The PISA data are cross-sectional but might help to inform research studies within countries.

In closing, it is important to mention that the creation and delivery of the innovative PISA CPS instrument included both simulated collaboration of a hard-to-measure construct (Scalise, 2012) and sharing of some process data. This was critical to the examination here, as has been the case for other collaboration-oriented assessments (Greiff et al., 2014, 2015, 2016). This analysis underscores that addressing challenges of education in the 21st century may continue to require new data sources, to address new challenges for education worldwide.

## Author Contributions

All authors listed have made a substantial, direct and intellectual contribution to the work, and approved it for publication.

## Conflict of Interest Statement

The authors declare that the research was conducted in the absence of any commercial or financial relationships that could be construed as a potential conflict of interest.

## Footnotes

1 www.oecd.org/pisa/data/
