# Supplementary material for: Collaborative Problem Solving: Processing Actions, Time, and Performance
Source: Front Psychol. 2019 Jun 7;10:1280. doi: 10.3389/fpsyg.2019.01280 (PMC6566913; doi:10.3389/fpsyg.2019.01280)
Supplement: Supplementary file 1 [file Presentation_1.pdf]

```
#read file from Paul code
#xandar=read.table(file="C:/Users/Paul/My Documents/CPS PISA/xandar.txt",header=TRUE)

#############read file select
db_irt1 = file.choose() #let's you browse to the location of a dataset and name the path as a variable
xandar <- read.table(file=db_irt1, header=TRUE) #reads in csv data set for the IRT exercise
View(xandar)
summary(xandar)
structure(xandar)
dim(xandar)

#############Paul Xandar analysis code
xan=xandar
xan[,4]=log(xandar[,4])
xan[,6]=log(xandar[,6])
xan[,8]=log(xandar[,8])
xan[,10]=log(xandar[,10])
xan[,3]=log(xandar[,3])
xan[,5]=log(xandar[,5])
xan[,7]=log(xandar[,7])
xan[,9]=log(xandar[,9])
View(xan)
write.csv(xan,"xan_Rescore.csv")

library(lavaan)
xandarmodel =  ' A =~ C1A + C2A + C3A + C4A
T =~ C1T + C2T + C3T + C4T
sum =~ sum1 + sum2 + sum3 + sum4
C1T ~ C1A
C2T ~ C2A
C3T ~ C3A
C4T ~ C4A
C1T ~ sum1
C2T ~ sum2
C3T ~ sum3
C4T ~ sum4
sum1 ~~ C1A
sum2 ~~ C2A
sum3 ~~ C3A
sum4 ~~ C4A
 '
estimate = cfa(model=xandarmodel, estimator="WLSMV",data=xan,std.lv=TRUE)
summary(estimate, standardized=TRUE, fit.measures=TRUE)

# Plot path diagram:
library(semPlot)
semPaths(estimate, title = FALSE, curvePivot = TRUE)
# Standardized parameters:
semPaths(estimate, "std", edge.label.cex = 0.5, curvePivot = TRUE)

##get cite for current version of lavaan
citation("lavaan") # here's the citation for your current version of lavaan
citation()

#ck for normality
#############read file select for clean data
db_irt1 = file.choose() #let's you browse to the location of a dataset and name the path as a variable
xandarNorm <- read.csv(file=db_irt1, header=TRUE, sep=",") #reads in csv data set for the IRT exercise
View(xandarNorm)
library(MVN)
# pull out just the variables we're using (x1-x9)
result = mvn(data = xandarNorm, univariatePlot = "histogram")

#get nicer tables
library(dplyr)
```

```
library(tidyr)
parameterEstimates(fit, standardized=TRUE) %>%
  filter(op == "=~") %>%
  select('Latent Factor'=lhs, Indicator=rhs, B=est, SE=se, Z=z, 'p-value'=pvalue, Beta=std.all) %>%
  kable(digits = 3, format="pandoc", caption="Factor Loadings")

#look at the residuals, keep an eye out for residual correlations larger than about .1
residuals(fit, type = "cor")$cor
cor_table <- residuals(fit, type = "cor")$cor

cor_table[upper.tri(cor_table)] <- NA # erase the upper triangle
diag(cor_table) <- NA # erase the diagonal 0's

kable(cor_table, digits=2) # makes a nice table and rounds everyhing to 2 digits
```
